# BGRS: bioinformatics of genome regulation and data integration

**DOI:** 10.1515/jib-2023-0032

**Published:** 2023-11-16

**Authors:** Yuriy L. Orlov, Ming Chen, Nikolay A. Kolchanov, Ralf Hofestädt

**Affiliations:** Institute of Cytology and Genetics, Siberian Branch of the Russian Academy of Sciences, 630090 Novosibirsk, Russia; Life Sciences Department, Novosibirsk State University, 630090 Novosibirsk, Russia; The Digital Health Institute, I.M. Sechenov First Moscow State Medical University of the Ministry of Health of the Russian Federation (Sechenov University), 119991 Moscow, Russia; Agrarian and Technological Institute, Peoples’ Friendship University of Russia, 117198 Moscow, Russia; Department of Bioinformatics, College of Life Sciences, Zhejiang University, Hangzhou 310058, China; Faculty of Technology, Bielefeld University, Bielefeld, Germany

Gene expression regulation is one of the priority topics for bioinformatics development. The topic was one of background research areas for the Journal of Integrative Bioinformatics [1, 2]. Experimental studies of gene regulation at transcription level led to the development of the databases on transcription factor regulation – TRRD and TRANSFAC [3]. The series of bioinformatics tools for the transcription factor binding sites prediction and integrations to the databases has been developed [4–6]. We have published papers on such tools following the rise of high-throughput sequencing technologies and corresponding growth of the experimental data [7, 8]. Integration of the bioinformatics resources and databases, standardization of data exchange became separate bioinformatics field [9, 10]. Contemporary machine learning and Big Data analysis methods serve to solve the same problems of gene expression regulation in much larger scale [4, 11, 12].

Here we review current trends in bioinformatics of gene expression based on the material discussed at BGRS\SB (Bioinformatics of Genome Regulation and Structure\Systems Biology) conference held in Novosibirsk biannually. Recent BGRS\SB meeting was hold in July 2022 (https://bgrssb.icgbio.ru/2022/). Previously we had special collection of the papers from the conference materials discussed at BGRS-2020 [2] and earlier BGRS events [13–15].

In year 2023, we celebrate the 25th anniversary of the Bioinformatics of Genome Regulation and Structure conference series since 1998. As the members of the conference Program committee, we are happy to remind the history of this great international bioinformatics conference. The first meeting of the “Bioinformatics of Genome Regulation and Structure/Systems Biology” (BGRS/SB) conference series was held in Novosibirsk in 1998, organized by the science team of Institute of Cytology and Genetics, Siberian Branch of the Russian Academy of Sciences and Conference Chairman Prof N. A. Kolchanov. At the first event just a small group of scientists gathered in Novosibirsk; they still remember exiting science atmosphere and hot discussions at Altai mountains post-conference trip in 1998 (https://conf.icgbio.ru/bgrs98/committee/). BGRS is the longest-running biannual bioinformatics conference series in Russia gathering scientists, bioinformaticians, mathematicians and IT-specialists, biophysicists, ecologists, medical doctors, and geneticists Novosibirsk (see the video about 20th anniversary at the conference web-site). It was grown from a relatively small bioinformatics meeting with less than 50 persons to large multi-conference with parallel sessions joining together up to 500 participants from different countries (https://bgrssb.icgbio.ru/2022/). Starting from classical bioinformatics at first meeting, the BGRS conference series includes sections and parallel events on systems biology, genomics, computational plant biology, evolution, medical genetics. Latest BGRS\SB-2022 event hosted 13 symposia and sections:

1. Symposium “Genomics, transcriptomics and bioinformatics”;
2. Symposium “Systems computational biology”;
3. Symposium “Structural biology and pharmacology: computational and experimental approaches”;
4. Symposium “Evolutionary, population and medical genomics/genetics of human”;
5. Symposium “Biotechnologies: computational and experimental approaches”;
6. Symposium “Genetics, bioinformatics and systems biology of plants”;
7. Symposium “Animal genetics, bioinformatics and systems computational biology”;
8. Symposium “Biomedicine, bioinformatics and systems computational biology”;
9. Symposium “Mathematics, bioinformatics and systems computational biology of COVID-19”;
10. Symposium “Cognitive sciences, neurogenetics, neuroinformatics and systems computational biology”;
11. Symposium “Systems biology and bioinformatics of DNA repair processes and programmed cell death”;
12. Section “Systems biology of aging: experimental and computational approaches”;
13. Symposium “Big genetic Data Analysis, deep learning, mathematical modeling and supercomputing”.

Each symposium description demands separate publication. First section of the multi-conference close to bioinformatics of gene expression regulation is “Genomics, transcriptomics and bioinformatics”. The topic on COVID-19 appeared at this section at the conference in 2020 when the BGRS-2020 passed in distant format. The coronavirus studies include database development [16]; now it is more focused on medical consequences of the coronavirus disease, and fundamental studies of pathogen evolution.

Note the satellite society meetings held in parallel to main BGRS event, such as Russian-German Virtual Bioinformatics Network, Sino-Russian workshops on bioinformatics [1, 17] (http://www.zjbioinformatics.org/meetings/2020/index.html; http://glab.hzau.edu.cn/subpages/RESOURCES/meeting.php). Current join international projects such as “Smart Crop” [6] raised from these workshops.

We’d like to commemorate the international science meetings on genetics organized by the Institute of Cytology and Genetics SB RAS, such as memorial Belyaev conference on genetics and evolution «Belyaev Readings-2017» (http://conf.bionet.nsc.ru/belyaev100/en) [18]. The full member of the USSR Academy of Sciences Professor Dmitry K. Belyaev (1917–1985) was the founder and first director of the Institute [19]. D.K. Belyaev was the outstanding scientist, evolutionist and geneticist who investigated the background on the genetics of behavior and the domestication of wild animals. The conference was devoted to genetics, discussing new trends in animal genetics based on next-generation sequencing technologies for understanding neurobiology mechanisms.

We have acknowledged fundamental research studies works conducted by Academician Prof. Vladimir K. Shumny on plant genetics [20], Prof. Vadim A. Ratner on mathematical biology [21–23], and Dr. Vitaly A. Likhoshvai on gene network theory [24, 25]. Mathematical biology and genetics were in foundation of bioinformatics in Novosibirsk since 1960s. Earlier theoretical works on mathematical biology by Prof. V.A. Ratner [21, 22] gave background for modern bioinformatics research.

The Systems Biology and Bioinformatics (SBB) School series in Novosibirsk were initially conceived as satellite event for young scientists held at the same time as BGRS\SB conference series, becoming now large independent event associated with Novosibirsk State University [26–28].

The BGRS conference series has a history of initiating successful Special Issues on bioinformatics in the bioinformatics journals. The recent examples include special issues at *Frontiers in genetics* and *MDPI International Journal of Molecular Sciences* on biomedical applications – “High-Throughput Sequencing-Based Investigation of Chronic Disease Markers and Mechanisms” [29], “Medical Genetics, Genomics and Bioinformatics” [30, 31], and on plant biology – special issue “Plant Biology and Biotechnology” [32]. We have presented special issues “Research Topics of the Bioinformatics of Gene Regulation” [33], and the series of the matical issues on genome regulation and systems biology at *Frontiers in Genetics* journal – Research Topic “Bioinformatics of genome regulation and systems biology” repeating the BGRS conference name and collecting the materials discussed at BGRS\SB meetings [34, 35]. Such wide range of journal issue refers to the topics and sessions organized in the frames of BGRS\SB multi-conference, confirming its actuality.

We had organized publications following the BGRS conferences in special journal issues at BioMed Central – *BMC Genomics* [13, 36], *BMC Genetics* [26], *BMC Medical Genomics* [27, 37], and *Journal of Bioinformatics and Computational Biology* [14, 15, 38] as well. The *Journal of Integrative Bioinformatics* supported this field by organization of special journal issues and promoting international collaboration on bioinformatics [2]. Thus, research on gene expression topic is in high demand.

In the special issue we present work by Haoyu Chao and co-authors [6] on plant bioinformatics raised from the joint Russian-Chinese research project on integrating omics data from crop plants (“Smart Crop” – Cognitive Platform for Reconstruction, Visualization and Analysis of Stress Response Networks based on ANDSystem and Multiomics in Rice and Wheat).

Evgeniya Antropova and colleagues [39] discuss gene network reconstruction based on ANDsystems tools [40].

To summarize and overview current trends note that main focus of the BGRS\SB-2022 conference was on the analysis of gene expression regulation in a genome on systems level, such as gene networks and systems biology models [5]. The necessity of community-wide collaboration is important for bioinformatics, as it was underlined in [11]. Linking of the metadata from the databases became a trend in integrative bioinformatics [40, 41]. New bioinformatics challenges are arising in the context of the COVID-19 consequences and viral genome studies by high-throughput techniques [42]. Finally, we note emerging topic of Big Data in medicine demanding development of new bioinformatics approaches [43].

Next BGRS meeting is scheduled for 2024. We aim to support the international collaboration in the frames of the BGRS conference by organization of new platforms such as special journal issue “25th Anniversary of the Bioinformatics of Genome Regulation and Structure conference series” (https://www.mdpi.com/journal/ijms/special_issues/0LGA6103S5).

Continuing series of international conferences covering gene expression research topic is the Congress of Russian Biophysicists. It is organized once in 4–5 years, recent VII Congress has sections on molecular biophysics and gene network models as well [44, 45].

We have additional research materials on medical bioinformatics that missed this BGRS-2022 special issue of the *Journal of Integrative Bioinformatics*. The related manuscripts will be published in the next regular issue.
